# A DNA vaccine targeting TcdA and TcdB induces protective immunity against *Clostridium difficile*

**DOI:** 10.1186/s12879-016-1924-1

**Published:** 2016-10-22

**Authors:** Bao-Zhong Zhang, Jianpiao Cai, Bin Yu, Yanhong Hua, Candy Choiyi Lau, Richard Yi-Tsun Tsun Kao, Kong-Hung Sze, Kwok-Yung Yuen, Jian-Dong Huang

**Affiliations:** 1 School of Biomedical Sciences, The University of Hong Kong, Li Ka Shing Faculty of Medicine, 3/F, Laboratory Block, 21 Sassoon Road, Pokfulam, Hong Kong China; 2 Department of Microbiology, The University of Hong Kong, University Pathology Building, Pokfulam, Hong Kong China; 3 HKU-Shenzhen Institute of Research and Innovation, The University of Hong Kong, Shenzhen, China; 4 The Centre for Synthetic Biology Engineering Research, Shenzhen Institutes of Advanced Technology, Chinese Academy of Science, Shenzhen, China

**Keywords:** DNA vaccine, *Clostridium difficile*, Toxin A (TcdA), Toxin B (TcdB)

## Abstract

**Background:**

*Clostridium difficile-*associated disease (CDAD) constitutes a great majority of hospital diarrhea cases in industrialized countries and is induced by two types of large toxin molecules: toxin A (TcdA) and toxin B (TcdB). Development of immunotherapeutic approaches, either active or passive, has seen a resurgence in recent years. Studies have described vaccine plasmids that express either TcdA and/or TcdB receptor binding domain (RBD). However, the effectiveness of one vector encoding both toxin RBDs against CDAD has not been evaluated.

**Methods:**

In the study, we constructed highly optimized plasmids to express the receptor binding domains of both TcdA and TcdB from a single vector. The DNA vaccine was evaluated in two animal models for its immunogenicity and protective effects.

**Results:**

The DNA vaccine induced high levels of serum antibodies to toxin A and/or B and demonstrated neutralizing activity in both in vitro and in vivo systems. In a *C. difficile* hamster infection model, immunization with the DNA vaccine reduced infection severity and conferred significant protection against a lethal *C. difficile* strain.

**Conclusions:**

This study has demonstrated a single plasmid encoding the RBD domains of *C. difficile* TcdA and TcdB as a DNA vaccine that could provide protection from *C. difficile* disease.

## Background

*Clostridium difficile* (*C. difficile*) is one of the most predominant pathogens causing nosocomial intestinal infections in industrialized countries. This bacterial species causes about 10–20 % of the cases of antibiotic-associated diarrhea, up to 70 % of the cases of antibiotic-associated colitis, and the vast majority of cases of pseudomembranous colitis. *Clostridium difficile-*associated disease (CDAD) causes economic loss of billions of US dollars in many industrialized countries [1–4]. There is an increasing incidence of CDAD in China caused by rapid economic development and the frequent use of antibiotics. CDADs are mainly caused by antibiotic-induced alteration of the normal flora of the intestine, particularly the long-term use of broad-spectrum antibiotics, thereby allowing *C. difficile* to proliferate. Cancer chemotherapeutics, hospitalization and immune deficiency are also risk factors, especially in the immunocompromised and the elderly [5]. The clinical manifestation of CDAD is complicated, ranging from being a symptomless carrier to contracting life-threatening pseudomembranous colitis. The prognosis of severe cases indicate that the chance of mortality is 40 %. Metronidazole and Vancomycin are the major treatment drugs for CDAD [6]. However, the *C. difficile* genome has been found to contain multiple-antibiotic resistant genes and *C. difficile* clinical isolates resistant to both Metronidazole and Vancomycin have been reported [7], which increase the difficulty for treatment of *C. difficile* in the future. For all these reasons, the design of vaccines against CDAD is very important.

Disease caused by *C. difficile* is due to two enteric toxins - TcdA and TcdB, produced by toxigenic strains [8–11]. TcdA is an enterotoxin with cytotoxic activity [12], whereas TcdB is a potent cytotoxin but has limited enterotoxic activity. TcdA and TcdB show considerable sequence and structural homology. Both have a C-terminal RBD and N-terminal glucosyltransferase enzymatic domain [13, 14]. Repeating sequences in the TcdA and TcdB genes harbor epitopes that can elicit toxin neutralizing antibodies. Many studies have proposed the RBD as a suitable target for a vaccine or immunotherapy [15–23].

Over the past two decades, great progress has been achieved in the vaccine development against CDAD [15, 17–19, 24–26]. However, most vaccine research for *C. difficile* target a single antigen, either TcdA or TcdB or a surface-layer protein (SLP) [24]. Furthermore, the incidence of A^-^B^+^ *C. difficile* strains appears to be increasing worldwide over the past decade. These strain types now represent a substantial number of *C. difficile* isolates. New therapeutic approaches for CDAD treatment such as toxin binders, passive immunotherapy or active immunization through vaccination will now need to target both TcdA and TcdB.

DNA vaccination is an effective platform to generate antigen-specific antibodies and cell-mediated immunity. The most prominent advantage of developing multivalent DNA vaccines is that a plasmid vector with multiple antigen epitopes can be cloned. Several other groups have described vaccine plasmids that express either TcdA and/or TcdB RBD against CDAD [15, 19]. In this study, we created a DNA vaccine of *C. difficile*, which encodes both toxin RBDs of *C. difficile*. The DNA vaccine was evaluated in two animal models for its immunogenicity, the ability to induce toxin neutralizing antibodies and in vivo anti-toxin protective immunity.

## Methods

### Plasmid design

**Fig. 1:**
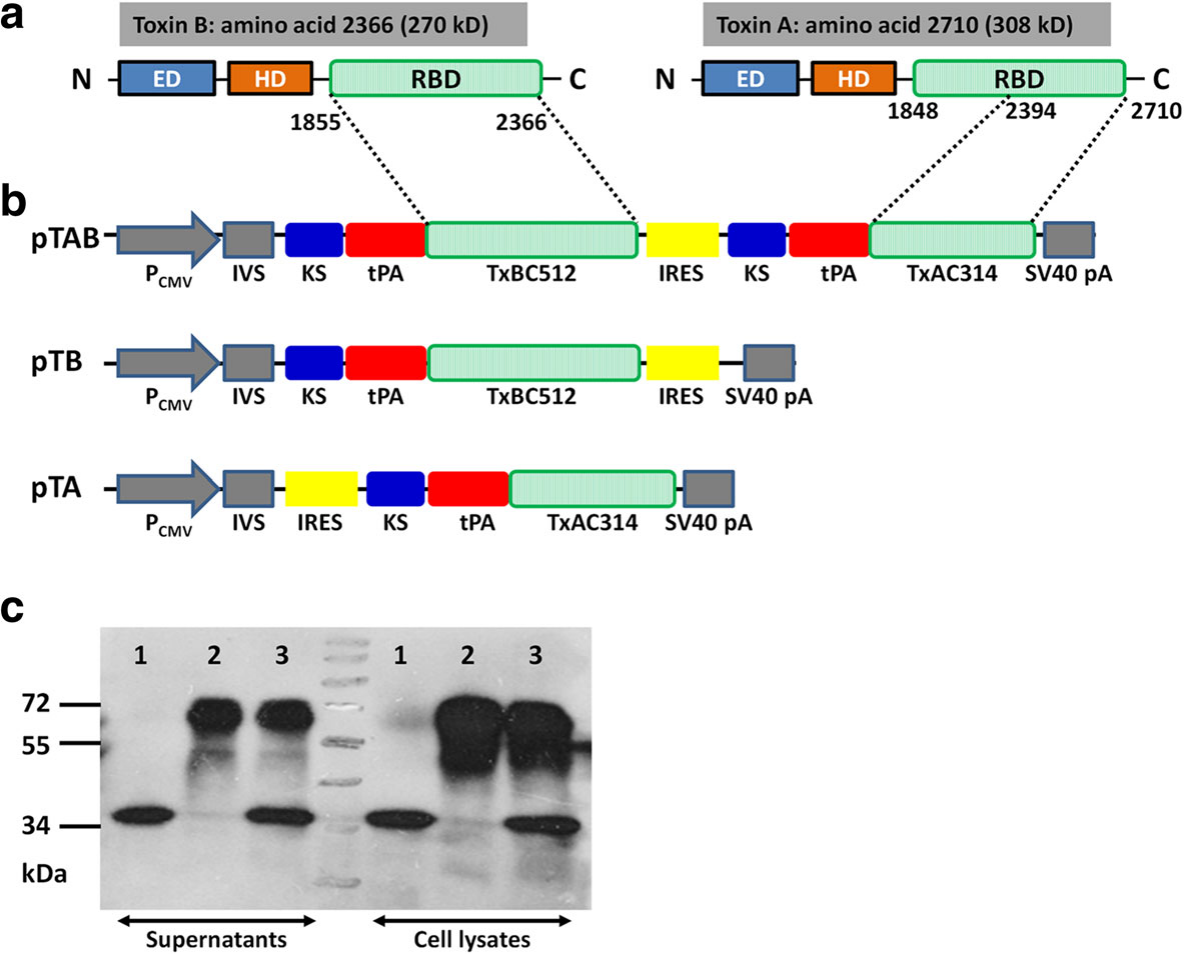
A schematic description of vaccine vector. **a** Linear depiction of the three major domains identified within *C. difficile* TcdA and TcdB. Note: ED; Enzymatic Domain; HD: Hydrophobic Translocation Domain; RBD: Receptor Binding Domain; IVS: Synthetic intron. **b** Schematic depiction of the vaccine gene sequence as inserted into the eukaryotic expression vector, pIRES. **c** Protein expression from vaccine vectors following transient transfection of COS7 cells. Immunoblot of COS7 cell lysates and supernatants following transient transfection with pTA (line1), pTB (line2) and pTAB (line3) for detection of expressed protein products. Supernatant was clarified at 18,000 × g for 30 min prior to the procedure. The expected size of TcdA-RBD is 35 kDa, TcdB-RBD is 60 kDa

The amino acid sequences corresponding to the RBD of *C. difficile* TcdA (strain ATCC 43255/VPI 10463, residue positions 2394–2710) and TcdB (strain ATCC 43255/VPI 10463, residue positions 1855–2366) were identified [13, 27]. A tissue plasminogen activator (tPA) sequence, Kozak sequence, and an initiation codon were incorporated as shown in Fig. 1. Following commercial synthesis (http://www.genscript.com/), these two genes were inserted into the commercial vector pIRES (Clontech Laboratories, Inc, USA). TOP10 chemically competent *E. coli* was transformed and positive clones confirmed by restriction digestion and DNA sequencing (BGI, China). The resulting three plasmids are referred to as (1) pTAB, (2) pTB and (3) pTA (Fig. 1b).

### Bacterial strains and growth conditions

The *C. difficile* strain BI/NAP1/027 is a gift from Dr. WC Yam, Queen Marry Hospital (Hong Kong). RBDs from the ATCC 43255/VPI10463 and the BI/NAP1/027 strain share highly conserved sequences. ATCC 43255 TcdA RBD has a 96.2 % identity with that of BI/NAP1/027, while there is an 88.5 % identity between the TcdB RBD sequences of BI/NAP1/027 and ATCC 43255. BI/NAP1/027 was cultured in Peptone Yeast Extract Agar or broth (Sigma-Aldrich) in an anaerobic atmosphere (10 % CO_2_, 10 % H_2_, 80 % N_2_) at 37 °C for overnight (OD 1.2; 2.5 × 10^8^ CFU/ml). The bacteria were harvested using endotoxin-free PBS, washed twice, and suspended in PBS at a concentration of 1 × 10^9^ CFU/mL.

### Protein expression

COS7 cells were plated in a 6-well dish at a density of 2 × 10^6^ cells per well in Dulbecco’s Modified Eagle’s Medium (DMEM) with 10 % Fetal Calf Serum (FCS) (v/v) and 2 % penicillin–streptomycin (v/v). 24 h post-plating, COS7 cells were transfected with 10 μg of DNA vaccine vectors (pTA, pTAB and pTB). At 48 h post-transfection, the cell lysates and supernatant were collected and stored at -80 °C. The supernatant was centrifuged at 16,000 × g for 45 min prior to Western blotting.

### Murine immunogenicity study

**Fig. 2:**
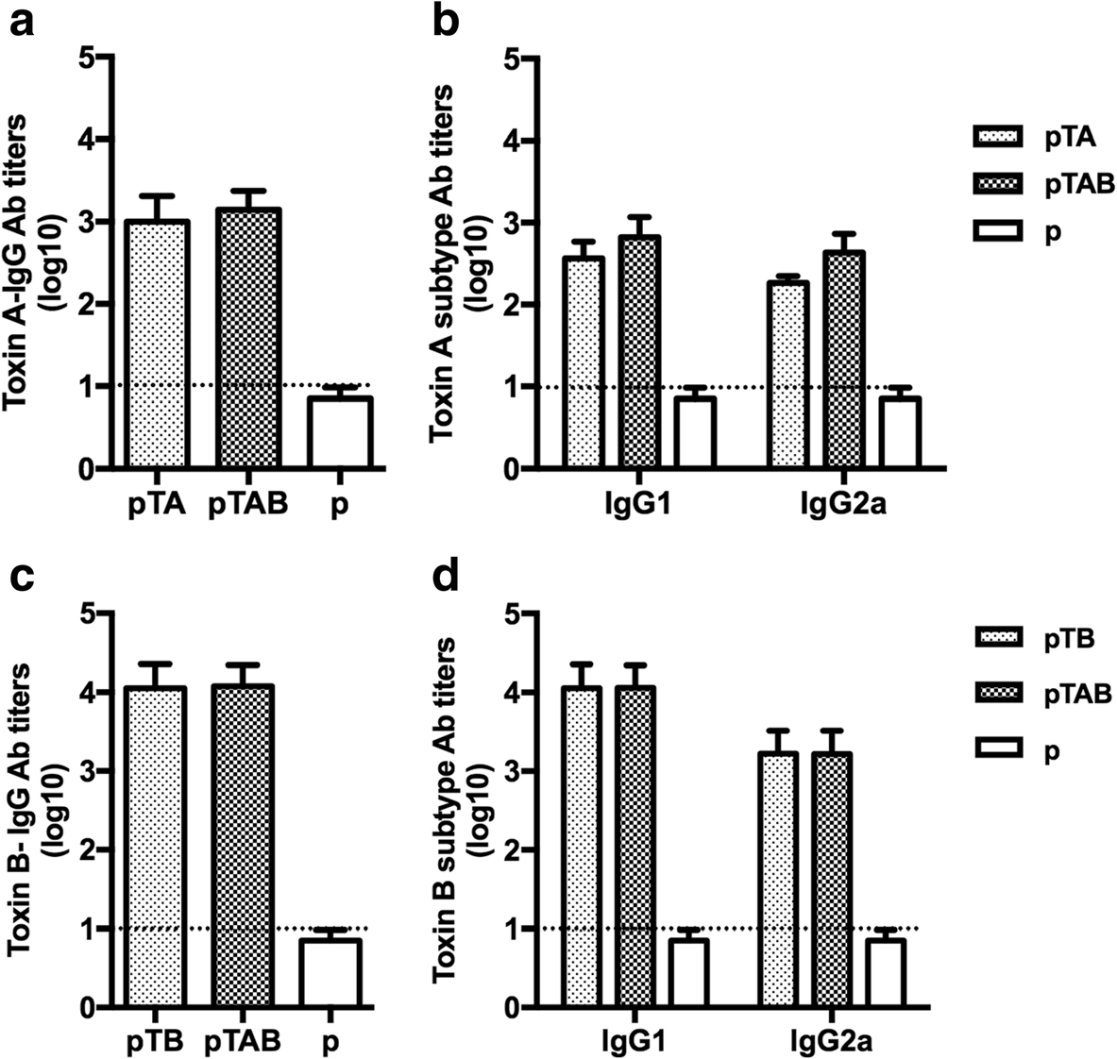
ELISA detection of anti-TcdA/B antibody titers. The data are expressed as geometric mean titer (GMT) of TcdA-specific antibody ± standard deviation (SD) of 10 mice per group. The lower limit of detection (1:10) is shown as dotted lines. The experiment was repeated at least twice. **a** TcdA-specific IgG antibody responses in mouse sera collected at 7 days after each vaccination. **b**: TcdA-specific IgG1 and IgG2a antibody responses in mouse sera obtained 7 days after the last boost. **c** TcdB-specific IgG antibody responses in mouse sera collected at 7 days after each vaccination. **d** TcdB-specific IgG1 and IgG2a antibody responses in mouse sera obtained 7 days after the last boost

Six-week-old female BALB/c mice (6 mice per group) were obtained from the Laboratory Animal Unit (LAU) of The University of Hong Kong (HKU) and housed in the animal room of Department of Microbiology. All mouse experiments were approved by the Committee on the Use of Live Animal in Teaching & Research (CULATR) of HKU (Approval No. 2596-11). To evaluate the immunogenicity of the DNA vaccine, LPS free (<100 IU) plasmid DNA for inoculation was extracted. Five groups of BALB/c were immunized at days 1, 14 and 28 (Fig. 2) with 50 μg of plasmid DNA by intramuscular injection (rear limbs). Groups were divided into immunizations by (1) pIRES alone; (2) pTA; (3) pTAB; (4) pTB; and (5) PBS as shown in Table 1. Each mouse experiment was repeated in two independent experiments. Blood samples were drawn by tail vein bleeding on days 0, 21, and 35 for immunologic evaluation. Mice were challenged with *C. difficile* TcdA or TcdB. Toxin challenge was performed by inoculating mice intraperitoneally (i.p) with 100 % of the minimal lethal dose (MLD) of the toxin. Mice were monitored for 14 days and survival was recorded for each vaccination group. The MLD of both toxins were confirmed by titration on age match control BALB/c mice. The MLD of toxins A and B were identified to be 50 ng and 25 ng, respectively.

**Table 1:** In vitro and vivo evaluation of toxin neutralizing antibody following immunization of mice with DNA vaccine

Vaccine^a^	Dose	Toxin neutralizing titer ED_50_^b^		Toxin challenge surival^c^		
		Anti-TcdA	Anti-TcdB	TcdA	TcdB	TcdA + B
p	50 μg	0	0	0	0	0
pTA		100 ± 30.15	0	90 %	0	0
pTB		0	1133 ± 327.9	10 %	100 %	0
pTAB		140 ± 27.63	1200 ± 294.4	100 %	100 %	80 %

^a^Mice received three immunizations (day 0, day 14 and day 28)

^b^Sera were obtained 7 days following the third immunization. The data are expressed as geometric mean toxin neutralizing titer ± Standard Error of the Mean (SEM) of 10 mice per group

^c^Balb/C mice (10 mice/group) were challenged i.p with 50 ng TcdA or/and 25 ng TcdB 10 days following the second boost of DNA immunization

### Hamster immunogenicity study

Golden Syrian adult female hamsters (6-week-old, weighing ∼ 100 g) were purchased from LAU of HKU and were housed individually in micro-isolator cages of Department of Microbiology. Hamster experiments were also approved by CULATR of HKU (Approve No. 2903-12). Hamsters were vaccinated by i.m. injection for three times in the thigh, on day 1, 14 and 28, with 100 μg pTAB, pTA or pTB, respectively. Controls were vaccinated with empty plasmid (pIRES, p). Serum samples were collected on days 21, and 35 for immunologic evaluation. On three consecutive days (day 36, 37 and 38), each hamster was treated with 10 mg/kg of clindamycin p.o. On day 39, each hamster received an intragastric challenge of 1x10^8^ CFU vegetative bacteria of *C. difficile* BI/NAP1/027. Hamsters were monitored at 12-h intervals. Each experiment was repeated in two independent experiments.

### ELISA

The TcdA and TcdB antibody titers were determined by enzyme-linked immunosorbent assay (ELISA). Briefly, TcdA and TcdB (0.5 μg/ml in 0.05 M carbonate/bicarbonate buffer, pH9.6, and 200 μL/well) were coated on ELISA plates (Nunc, Roskilde, Denmark) by incubation overnight at 4 °C. Plates were then blocked with PBS-5 % (w/v) non-fat milk for 3 h at 37 °C and washed for 4 times with 0.05 % Tween in PBS. Two-fold serially diluted mice sera were then added into the wells and incubated for 1 h at 37 °C. Plates were then washed 6 times with PBS-0.05 % Tween and incubated with HRP-conjugated goat anti-mouse IgG/IgG1/IgG2a for 1 h at 37 °C. Color was developed by using Trimethyl Borane (TMB) solution (Sigma) and absorbance was measured using an ELISA reader at 450 nm. The end-point serum antibody titers represent the reciprocal dilution of the last dilution providing an O.D. 2.1-fold higher than the O.D. of negative controls at the lowest performed dilution. A sample of pre-immune serum obtained from mice and hamsters were used as a negative control.

### Neutralizing antibody test

Toxin neutralizing titers of the antiserum were determined by using Vero cells and both toxins. Vero cells were grown in Eagle’s Minimum Essential Medium (EMEM) containing 10 % fetal calf serum. For neutralizing antibody test, 0.5 ng (100 μl) TcdA or 0.1 ng (100 μl) TcdB was incubated with 100 μl serial dilutions of serum obtained from immunized mice or hamsters. After mixing the antiserum and toxin at 37 °C for 90 min, the mixtures were added to 96 well plates containing 1x10^5^ Vero cells, and the plates were incubated in 5 % CO_2_ at 37 °C for 24 h. Incubation of Vero cells with toxin resulted in a loss of cell adherence and a change in cell morphology, which was detected by methyl thiazolyl tetrazolium staining of toxin treated Vero cells after discarding the non-adherent cells. The plates were read on a microtiter plate reader at a wavelength of 490 nm. The neutralization titer of an antiserum was recorded as the serum dilution which gives a 50 % reduction in toxin activity (ED50).

### Statistical analysis

Log-rank (Mantel-Cox) analysis was used to analyze the statistical significance of the data from the lethal challenge experiment. Analyses were performed using GraphPad Prism 5 (GraphPad Software, United States) and a *p*-value of < 0.05 was determined to indicate statistical significance.

## Results

### Protein expression

Supernatants and cell lysates were harvested at 48 h post-transfection and detected for protein expression via Western blotting with anti-His antibody. The target proteins were highly expressed in the supernatants of the cell lysates (Fig. 1c).

### Immunogenicity of the DNA vaccine in mice

To investigate the antibody titers of TcdA-specific and TcdB-specific antibodies in the sera of immunized mice, the levels of IgG, IgG1, IgG2a antibodies were detected by ELISA 7 days after the third immunization. As shown in Fig. 2a, toxin A-specific IgG antibodies were detected in mice immunized with pTA and pTAB, with mean titers of 1.0 × 10^3^ and 1.6 × 10^3^ respectively. High levels of TcdB-specific IgG antibodies were also detected in mice immunized with pTB and pTAB, both reaching 2.56 × 10^4^ (Fig. 2c).

To further observe IgG antibody subtype responses, TcdA and TcdB - specific IgG1 and IgG2a antibody titers were also detected. As shown in Fig. 2b, immunization with pTA and pTAB induced both Th1-(IgG2a) and Th2-(IgG1) associated TcdA-specific antibody responses with end point IgG1 and IgG2a antibody titers of 8.0 × 10^2^ and 2.0 × 10^2^ for pTA and 1.6 × 10^3^ and 8.0 × 10^2^ for pTAB, respectively. Similar with the anti-TcdB total IgG production, mice immunized with pTB and pTAB also induced both Th1-(IgG2a) and Th2-(IgG1) associated high levels of TcdB-specific antibody responses, IgG1 and IgG2a antibody titers up to 2.56 × 10^4^ and 3.2 × 10^4^ for pTB and pTAB, respectively (Fig. 2d). Furthermore, vaccination with empty vector alone induced only background level of TcdA or TcdB-specific IgG, IgG1 and IgG2a antibodies at the lower limit of detection (1:10). These results indicate that pTA, pTB and pTAB can induce both Th1-(IgG2a) and Th2-(IgG1) associated antibody responses.

To test the functional activity of the DNA vaccine induced antibodies to neutralize native toxin proteins, toxin neutralization tests were performed (Table 1). Serum samples from the pTAB group were found to have TcdA and TcdB neutralizing activity. Additionally, in mice immunized with pTA (pTB), the serum antibodies can also neutralize TcdA (TcdB) activity.

**Fig. 3:**
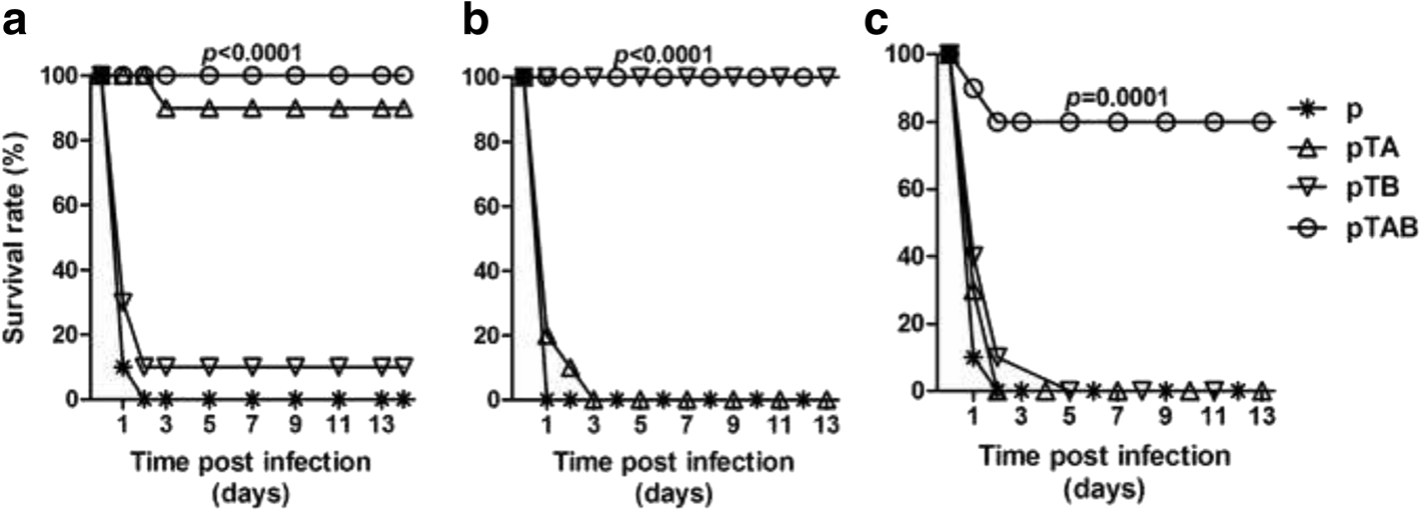
Survival in vaccinated Balb/C mice following challenge with purified. *C. difficile* toxins. Balb/C mice (10 mice/group) were challenged i.p with (**a**) 50 ng TcdA; (**b**) 25 ng TcdB; or (**c**) 50 ng TcdA and 25 ng TcdB. 10 days following the second boost DNA immunization, and monitored for survival for 14 days. *, *P* ≤ 0.05; ***, *P* ≤ 0.001. Data from two replicate experiments are shown

**Fig. 4:**
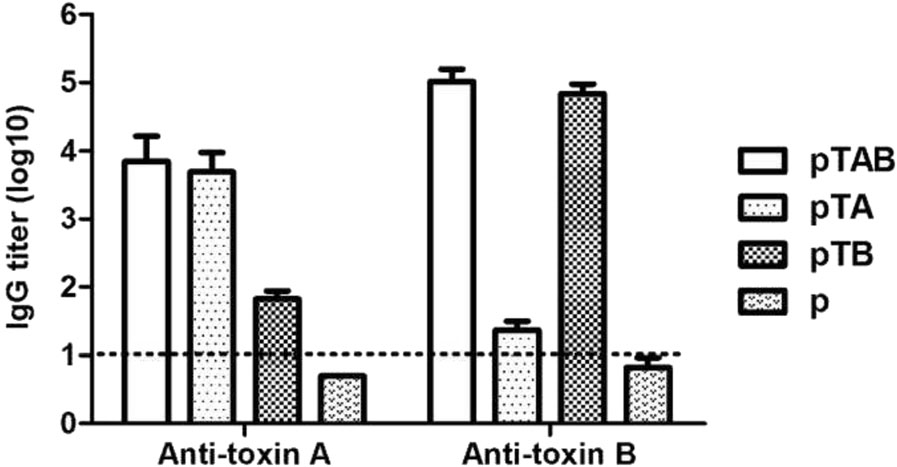
ELISA detection of hamster serum antibody titers induced by pTA, pTB and pTAB. The data are expressed as geometric mean titer (GMT) of TcdA or TcdB-specific antibody ± standard deviation (SD) of 10 mice per group

Toxin challenge mouse model was used to evaluate the protective efficacy of DNA vaccine. Immunized Balb/C mice were injected i.p. with 50 ng TcdA or/and 25 ng TcdB 10 days following the second boost of DNA immunization. Following TcdA challenge, 100 % (10/10) of the mice in the p group and 90 % (9/10) in the pTB group died (Fig. 4a). But for the pTAB and pTA groups, 100 and 90 % of the mice survived the lethal TcdA challenge, respectively (Fig. 3a). In the p and pTA group, all mice died following TcdB challenge (Fig. 4b). In contrast, 100 % pTAB and pTB immunized mice survived the lethal TcdA challenge (Fig. 3b). The result (Fig. 3c) shows that the pTAB had significantly improved the survival of the mice following TcdA plus TcdB challenge. Survival rate of 80 % was observed in mice immunized with pTAB. However, in the p, pTA and pTB group, all mice died within 2 days after TcdA plus TcdB challenge.

### Protective efficacy of pTAB in a hamster model

Hamster model is the gold standard for evaluation of vaccine against CDAD which can be induced by clindamycin following a challenge with *C. difficile.* To evaluate the protective efficacy of the DNA vaccine, hamsters (*n* = 6) were vaccinated for three times. Serum samples obtained 7 days following each immunization were checked by ELISA to detect antibodies. Significant levels of anti-TcdA and TcdB antibodies were detected after the third immunization by pTAB. Only background (titers < 10) were observed in empty vector immunized controls. As observed in the mice immunogenicity study, hamster anti-TcdB titers (pTAB: GMT = 1.0 × 10^5^, pTB: GMT = 6.8 × 10^4^) were consistently higher than those observed for anti-TcdA (pTAB: GMT = 7.1 × 10^3^, pTA: GMT = 4.9 × 10^3^) (Fig. 4).

**Fig. 5:**
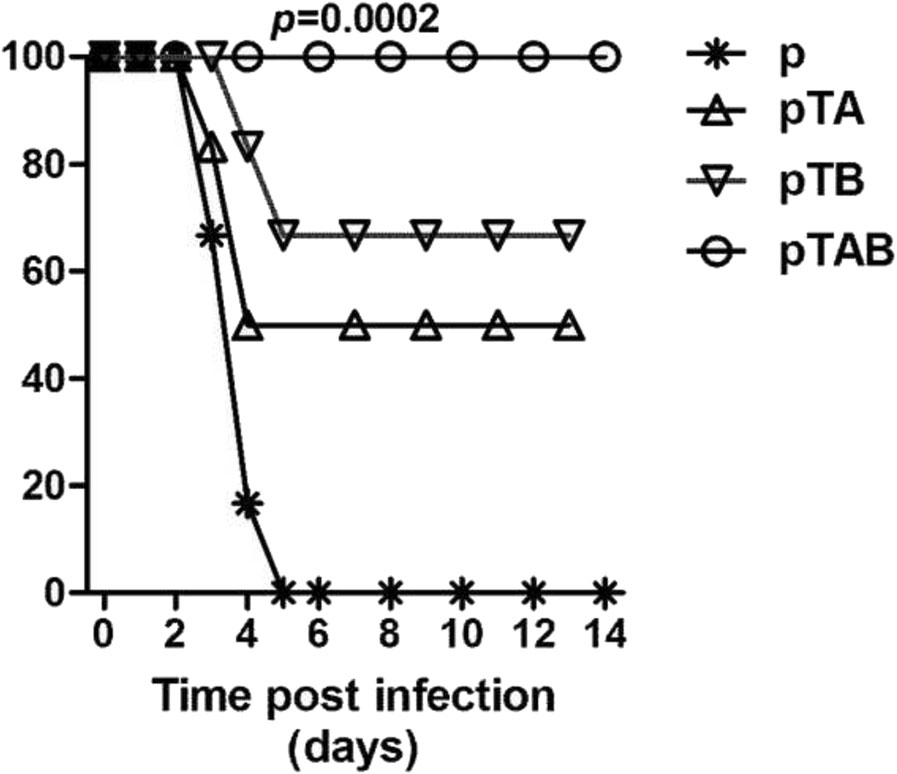
Survival in vaccinated hamsters following challenge with *C. difficile* BI/NAP1/027**.** Hamsters (*n* = 6) were immunized with the DNA vaccine. Two weeks following the third immunization, hamsters were treated with clindamycin p.o. (10 mg/kg) and the following day received an intra-gastric challenge of 10^8^ CFU *C. diff* BI/NAP1/027. *, *P* ≤ 0.05; ***, *P* ≤ 0.001. Data from two replicate experiments are shown

To evaluate in vivo protective efficacy, 8 days following the third immunization, hamsters were treated with clindamycin and challenged with 1 × 10^8^ CFU *C. difficile* BI/NAP1/027. Similar to the BALB/c mice model, the DNA vaccine group has significantly improved survival following 1 × 10^8^ CFU *C. difficile* BI/NAP1/027 challenge. Survival rate of 100, 50 and 66.7 % were observed in hamster immunized with pTAB, pTA and pTB respectively (Fig. 5). Specifically, CDAD was detected in 30 % of the empty vector immunized hamsters within 24 h. At 48 h, almost all hamsters in the group showed moderate to severe disease and had all died by day 5. However, hamsters immunized with pTAB did not have signs of CDAD until 60 h. The disease noted in pTAB group was less severe and 70 % of the hamsters recovered to normal health by day 6 (data not shown). Of most concern, 100 % survival was observed in the pTAB group at day 14. Additionally, all hamsters in pTAB group exhibited mild to moderate CDAD in the early stages of the experiments and remained symptom free at the end of the study.

## Discussion

*C. difficile* secretes two toxins: TcdA (both an enterotoxin and cytotoxin) and TcdB (a potent cytotoxin). These two toxins can mediate the pathogenesis of CDAD. Since 2002, researches have isolated a novel epidemic typed BI/NAP1/027 strain [28], which produces 16-fold higher level of TcdA and 24-fold higher level of TcdB than the nonhypervirulent strain VPI 10463 [8]. The role of TcdA and TcdB in CDAD has been confirmed in numerous studies. According to an early study in which two purified toxins were administered by intragastrically, CDAD was only detected after the administration of purified TcdA, and TcdB cannot induce severe disease unless it is co-administered with TcdA, suggesting that TcdA is the primary pathogenic factor and the toxins act synergistically [29]. After outbreaks of A^-^B^+^ pathogenic *C. difficile* variants, an important role for TcdB in *C. difficile* pathogenicity was established [30–32]. Since both toxins have the same importance to the pathogenesis of *C. difficile*, any immunotherapeutic drugs must target both TcdA and TcdB.

In this study we describe a DNA vaccine - pTAB, consisting of the TcdA RBD (15 of the 31 repetitive oligopeptide sequences) and the TcdB RBD (23 of the 24 repetitive oligopeptide sequences) joined by IRES sequence. This DNA vaccine candidate focuses on the TcdA and TcdB RBD portions. This choice is supported by previous research demonstrating that: (i) antibodies targeting the RBD of both TcdA and TcdB have toxin neutralizing activity [19, 33]. (ii) the passive transfer of anti-TcdA-RBD or TcdB-RBD antibodies are protective in animal CDAD models and [23] (iii) hamsters immunized with RBD of TcdA and/or TcdB are protected against CDAD [17].

Several other groups have described vaccine plasmids that express either TcdA and/or TcdB RBD [15, 16, 19]. In this report, we describe a new vaccine plasmid pTAB, that expresses both toxin RBD sequences. The pTAB was constructed from a commercial mammalian expression vector, pIRES, that allows high level expression of two genes of interest from the same bicistronic mRNA transcript. The vector contains the encephalomyocarditis virus (ECMV) internal ribosome entry site (IRES) flanked by two multiple cloning sites (MCS A and B), an arrangement that allows cap-independent translation of the gene cloned into MCS B [25]. In the DNA vaccine, TcdB-RBD and TcdA-RBD was cloned into the MCS A and MCS B, respectively. The pTAB vaccine plasmid is much more cost-effective than creating two separate plasmids.

Since antibody responses to both RBDs are important for control of CDAD. When DNA vaccine plasmids expressing either A-RBD or B-RBD are co-delivered, it seems that A-RBD dominates the immune response suggesting antigen interference [15]. The pTAB was designed in a way that the A-RBD was placed downstream of a partially disabled IRES sequence [25] to reduce the rate at which the TcdA-RBD is translated relative to that of TcdB-RBD, thus provoking the generation of a higher titer of B-RBD antibody compared to A-RBD. Immunization of mice and hamsters elicited the generation of both TcdA and TcdB antibodies (Figs. 2, 3 and 4) which were capable of neutralizing toxin in vivo assays (Table 1). Immunization with pTAB provided 100 % protection against a TcdA or TcdB MLD challenge, and also produced 80 % protection against TcdA MLD plus TcdB MLD challenge.

pTAB also demonstrated protective efficacy in the hamster CDAD model, reducing the severity and time till onset of CDAD and significantly protecting the hamster from mortality induced by challenge with BI/NAP1/027. The significance of this hamster model is characterized by a very rapid progression of CDAD and high mortality. In the empty vector group, hamsters challenged with 10^8^ CFU BI/NAP1/027 had all died by day 5, but 100 % survival was observed in the pTAB group at day 14. While hamsters in the pTAB group that exhibited CDAD were characterized as mild to moderate, all recovered and were symptom free by the end of study (14 days). Since BI/NAP1/027 is a double positive (A^+^B^+^) strain, immunized pTA and pTB cannot exhibit a 100 % survival.

Although recently one research has shown that two DNA plasmids encoding the TcdA and TcdB RBDs respectively can induce protective antibody responses if used together in mouse model, the study did not investigate whether a single-plasmid (pARBD or pBRBD) can protect mice from TcdA plus TcdB challenge [15]. Our experiments showed that neither pTA nor pTB single immunization was sufficient to protect mice from a TcdA plus TcdB MLD double challenge. In contrast, compared to the prior study, our research utilized one plasmid (pTAB) instead of two separate plasmids for immunization, achieving even better protection after challenged with double lethal dose toxins in mouse model.

## Conclusions

This study has demonstrated a single plasmid encoding the RBD domains of C. difficile TcdA and TcdB as a DNA vaccine that could provide protection from C. difficile disease.

## Abbreviations

*C. difficile*: *Clostridium difficile*
CDAD: *Clostridium difficile-*associated disease
CULATR: Committee on the Use of Live Animal in Teaching & Research
DMEM: Dulbecco’s Modified Eagle’s Medium
ED50: 50 % reduction in toxin activity
ELISA: Enzyme-linked immunosorbent assay
EMEM: Eagle’s Minimum Essential Medium
FCS: Fetal Calf Serum
LAU: Laboratory Animal Unit
MLD: Minimal lethal dose
RBD: Receptor binding domain
SLP: Surface-layer protein
TcdA: Toxin A
TcdB: Toxin B
TMB: Trimethyl Borane
tPA: Tissue plasminogen activator
